# A prospective cohort study of safety and patient satisfaction of voluntary medical male circumcision in Botswana

**DOI:** 10.1371/journal.pone.0185904

**Published:** 2017-11-07

**Authors:** Kathleen E. Wirth, Bazghina-werq Semo, Lisa P. Spees, Conrad Ntsuape, Scott Barnhart, Jenny H. Ledikwe

**Affiliations:** 1 Botswana International Training and Education Center for Health (I-TECH), Gaborone, Botswana; 2 Department of Epidemiology, Harvard T.H. Chan School of Public Health, Boston, MA, United States of America; 3 Department of Immunology and Infectious Diseases, Harvard T.H. Chan School of Public Health, Boston, MA, United States of America; 4 Department of Global Health, University of Washington, Seattle, WA, United States of America; 5 Cecil G. Sheps Center for Health Services Research, University of North Carolina at Chapel Hill, Chapel Hill, NC, United States of America; 6 Department of HIV/AIDS Prevention and Care, Botswana Ministry of Health, Gaborone, Botswana; Cardiff University, UNITED KINGDOM

## Abstract

Randomized trials have shown that voluntary medical male circumcision (VMMC) significantly reduces the risk of HIV acquisition in men. However, the rate of complications associated with the surgical procedure varies from 0.7% to 37.4% in real-world settings. We assessed the frequency, type and severity of adverse events following VMMC among 427 adult men surgically circumcised in southeastern Botswana; 97% completed ≥1 follow-up visit within seven days post-circumcision. Thirty moderate AEs were observed in 28 men resulting in an overall AE rate of 6.7%. Patient satisfaction was high: >95% were very or somewhat satisfied with the procedure and subsequent follow-up care.

## Introduction

Three randomized controlled trials demonstrated that male circumcision, when performed in a clinical setting by a trained provider, safely and significantly reduces the risk of heterosexually-acquired HIV infection in men [1–3] leading to a 2007 endorsement by the World Health Organization (WHO) of voluntary medical male circumcision (VMMC) as an add-on strategy for HIV prevention. The rate of moderate or severe adverse events (AE) following circumcision ranged from 0.8% to 3.6% in the two randomized trials conducted in Kenya and Uganda, respectively [1–2]; the South African trial reported an overall AE rate of 3.5%, inclusive of mild events [3]. Following the WHO’s endorsement, circumcision services have been rolled out in more than 14 countries across eastern and southern Africa [4]. A 2012 systematic review of 10 safety studies conducted across sub-Saharan Africa reported an overall pooled AE rate of 2.3%. However, there was significant heterogeneity: circumcision-associated AE rates ranged from <1% in Uganda [5] to >30% in South Africa [6–8].

In Botswana, similar to other VMMC programs in sub-Saharan Africa, monitoring and evaluation of VMMC-related AEs relies on a passive surveillance system. Although the system uses a standardized approach for assessing and recording complications, it is in place only in facilities that regularly provide circumcision services, and patients experiencing complications following VMMC may seek care at any healthcare facility. If the facility does not routinely provide circumcision services, clinic staff may lack sufficient training and/or clinical experience to appropriately identify and treat complications. Furthermore, complications treated at these facilities will not be captured in the surveillance system. Finally, patient completion of follow-up care during the wound-healing period can be very poor. Among more than 5,100 males circumcised in KwaZulu Natal, South Africa between 2011 and 2013, less than half were seen at the seven-day post-operative visit [9]. This is especially concerning given recent evidence from Kenya which suggested that among patients who do not return for follow-up care, the moderate/severe AE rate was approximately double that observed among patients who did return for follow-up care [10].

We sought to actively assess circumcision-associated AEs by prospectively following a cohort of men undergoing surgical VMMC at two government-run clinics in southeastern Botswana.

## Materials and methods

### Study design

This prospective cohort study was designed to assess (a) the frequency, type and severity of adverse events following VMMC; (b) prevalence and correlates of re-initiation of sexual activity and; (c) changes in risky sexual behavior following VMMC. The study was conducted by the International Training and Education Center for Health (I-TECH), a collaboration between the University of Washington and University of California, San Francisco. Ethical approvals were obtained by the Health Research and Development Committee at the Botswana Ministry of Health (MOH) and the University of Washington Institutional Review Board. Recruitment and enrollment of study participants occurred before undergoing VMMC but after individuals completed group education about the risks and benefits of VMMC, received individual counseling with clinic staff (including HIV testing) and provided written, informed consent for the procedure. Neither the pre-procedure activities described above nor the procedure itself were performed by study staff. A complete description of Botswana’s National Safe Male Circumcision program, including details on the procedure itself, can be found elsewhere [11].

### Subjects

Adult men undergoing VMMC through the National Safe Male Circumcision program were enrolled between November 2013 and April 2015 at two government-run clinics providing free circumcision services in Gaborone, the capital city of Botswana. Participant eligibility criteria were age 18 to 49 years, residence within 25 km of Gaborone, ever had sexual intercourse and documented HIV-negative test result. All participants provided written informed consent for participation in the study in addition to the consent obtained by clinic staff for the circumcision procedure.

### Data collection

After circumcision, study staff reviewed the registry for complications occurring during the surgery and performed a physical examination to assess post-procedure bleeding and other immediate adverse events. Post-procedure visits at two days, seven days, six weeks and three months were scheduled as outlined by the Botswana MOH guidelines for adult VMMC. A follow-up visit at 12 months was planned to coincide with annual HIV testing as per standard of care in Botswana. Each study participant was provided with a wallet-size reminder card noting the date of each follow-up visit. In advance of each visit, study staff telephoned participants to remind them of the upcoming visit. At each follow-up visit, study staff performed a physical examination, including inspecting the circumcision site and assessing for signs of STIs. Participants received BWP100 (approximately USD$8 at study initiation) at each post-operative visit as compensation for their time and travel costs. In the event of a missed visit, study staff made telephone calls to reschedule the appointment. If participants were unable to be reached by telephone after three attempts, study staff them at home.

The current analysis is restricted to data collected at two and seven days post-circumcision because the majority of adverse events occur during or immediately following VMMC and to ensure comparability of our results to previously-published safety studies conducted in other settings [12–15]. Participants were asked to complete a brief questionnaire about wound care, patient satisfaction and resumption of normal activities or work. Study data were collected and managed using REDCap (Research Electronic Data Capture), a secure, web-based application designed to support data capture for research studies hosted at the Institute for Translational Health Sciences at the University of Washington. [16].

### Outcomes

The primary outcomes of the current analysis were (1) frequency, type and severity of short-term adverse events following VMMC; (2) participant satisfaction with the procedure and follow-up care by clinic staff and (3) time to resumption of normal activities. We defined the types and severity of adverse events potentially related to VMMC prior to study initiation based on WHO guidelines for the recognition and management of post-circumcision complications [17] and included severity classifications tailored to each event type. The clinical assessment tool containing all event types and severity classifications is provided as supporting information (S1–S4 Appendices). Participant satisfaction with the procedure itself and the quality of follow-up care received was assessed using a four-level Likert-type scale with the following responses: very satisfied, satisfied, dissatisfied and very dissatisfied. Finally, we assessed time to resumption of normal activities with the following questions administered at each follow-up visit: “Have you resumed normal activities or work since the circumcision procedure?” and “How many days after the circumcision procedure did you resume normal activities or work?”

### Statistical analysis

The AE rate at two and seven days post-circumcision was calculated as the number of participants with ≥1 moderate or severe adverse events divided by the number of participants assessed at the respective visit. Pain-related events were not included in the non-type-specific AE rates because they often are associated with another complication [18]. The overall AE rate (i.e. combining observations at both follow-up visits) was defined as the number of distinct participants with one or more events at one or both follow-up visits divided by the number of participants assessed at one or both follow-up visits. We note that this approach ensures that participants who experienced an adverse event at both follow-up visits contributed only once to the calculation as well as participants who may have suffered from more than one adverse event during a follow-up visit. All analyses were conducted using SAS software version 9.4 (SAS Institute, Cary, NC).

## Results

**Fig 1:**
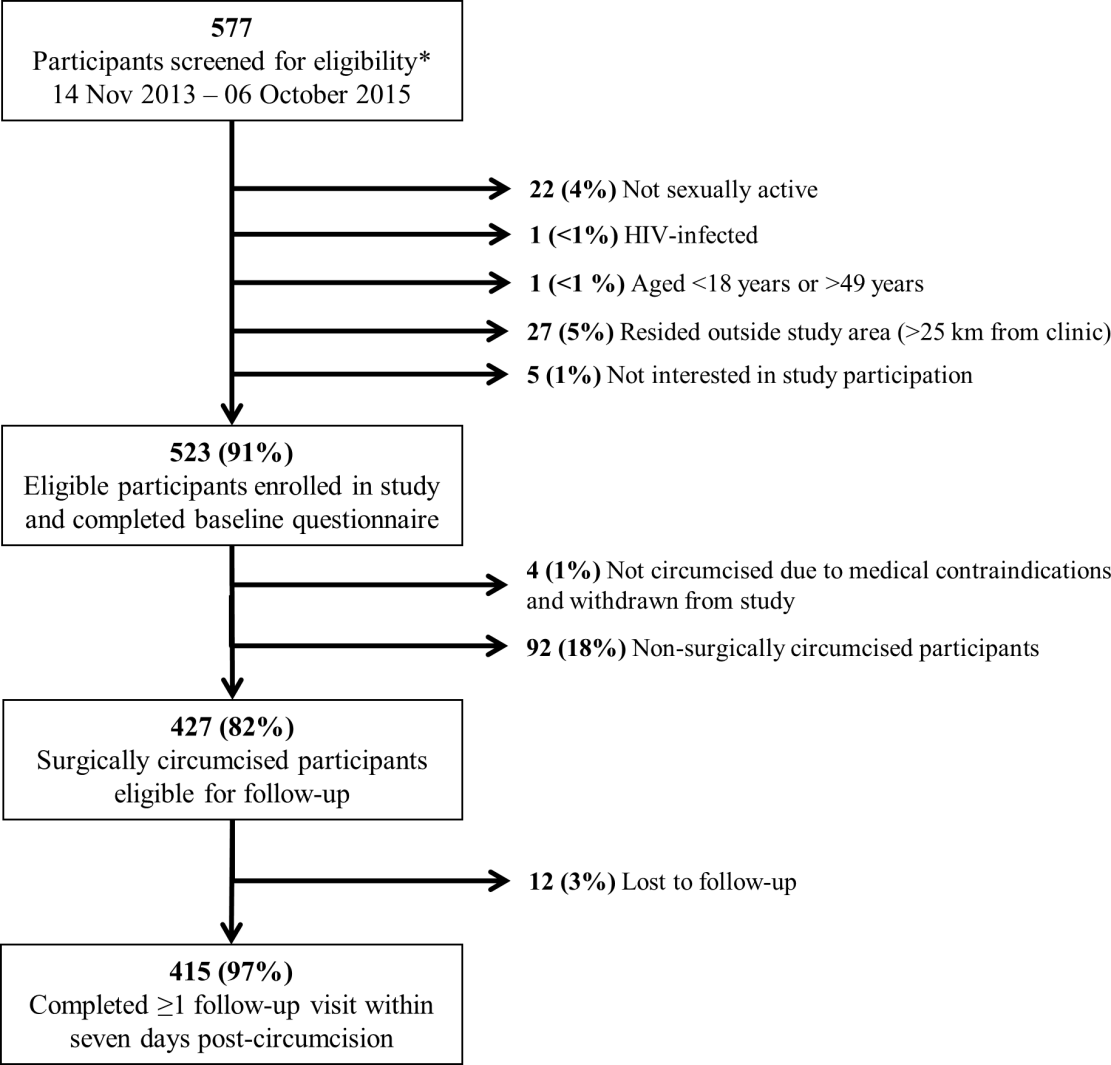
Flow chart of N = 577 men screened for circumcision eligibility within the National Safe Male Circumcision program in Gaborone, Botswana. Two participants did not meet two eligibility criteria and thus the number and percentages presented for individual reasons for ineligibility sum to >100%.

Between November 2013 and October 2015, research staff screened 577 men preparing to undergo VMMC for study participation (Fig 1).

A total of 528 (92%) participants were determined to be eligible for participation and 523 (99%) subsequently enrolled. Reasons for ineligibility included not sexually active (4%), residence outside of the area (5%), HIV-infection (<1%) and age <18 years or >49 years (<1%). Four individuals who met study eligibility criteria and consented to study participation were not circumcised due to medical contraindications that were identified prior to the procedure. Ninety-two participants were non-surgically circumcised with the PrePex device. Of the 427 surgically circumcised participants, 415 (97%) completed one or both follow-up visits at two and seven days post-circumcision (Fig 1). Three hundred eighty-one participants attended both visits.

Median (25^th^, 75^th^ percentile) age of study participants was 27 years (23, 31). Most men reporting being in a dating relationship but not living with that partner (53%); only 31 men (7%) were currently married. A minority of participants completed less than secondary education (4%), and most reported being currently employed (70%).

We observed a total of 31 non-pain-related moderate AEs among 28 men resulting in an overall AE rate of 6.7% (Table 1). One participant had an event at day 2 and a different event at day 7; two subjects had two events each at day 7 and no events observed at day 2 were still ongoing at day 7. All AEs were immediately treated by clinic staff at the time of the follow-up visit. Study staff followed up with all participants to ensure appropriate and timely resolution of any adverse event and documented receipt of any interventions (e.g. provision of acetaminophen, antibiotics) provided by clinic staff. One pain-related severe AE was observed two days post-circumcision. The participant reported a 9 of 10 on the pain scale, stating that his pain was severe enough that it woke him up the previous night. Upon further inquiry by clinic staff, he indicated that 500 mg of Paracetamol (acetaminophen) sufficiently alleviated his symptoms. The most common type of event reported was hematoma (2.7%, N = 12), followed by infection (2.2%, N = 9) and bleeding (1.2%, N = 5) (Table 1). Other AEs assessed but not observed included injury to the penis, excessive skin removed, micturition, and anesthesia-related complications. While no participants reported sexual intercourse in the first two days after the procedure, one participant reported engaging in sexual intercourse at his day 7 follow-up visit. This patient also had a mild hematoma at the time of this visit.

**Table 1:** Moderate/severe adverse events observed overall and 2 and 7 days post-circumcision among N = 427 HIV-uninfected, sexually-active adult men surgically circumcised within the National Safe Male Circumcision program in Gaborone, Botswana.

	2 days post-circumcision (n = 401)			7 days post-circumcision (n = 395)			Overall (n = 415)^a^		
	Events	Participants with ≥1 event	Rate per 100 circumcisions^b^	Events	Participants with ≥1 event	Rate per 100 circumcisions^b^	Events	Participants with ≥1 event^c^	Rate per 100 circumcisions^b^
Any adverse event^d^	10	10	2.5	21	19^e^	4.8	31	28^e^	6.7
Hematoma	4	4	1.0	8	8	2.0	12	11	2.7
Bleeding	3	3	0.7	2	2	0.5	5	5	1.2
Infection	2	2	0.5	7	7	1.8	9	9	2.2
Pain	1	1	0.2	3	3	0.7	4	4	1.0
Insufficient skin removed	1	1	0.2	1	1	0.2	2	2	0.5
Delayed healing	0	0	0	3	3	0.7	3	3	0.7

^a^ 415 participants attended one or both follow-up visits at day 2 or day 7.

^b^ Rate per 100 circumcisions calculated by dividing the number of participants assessed at the follow-up by the number of participants with ≥1 event

^c^ Number of unique participants with either ≥1 events at day 2 or day 7.

^d^ Exclusive of pain-related adverse events. Other adverse events assessed, but no events observed included injury to penis, excess skin removed, micturition, and anesthesia-related complications.

^e^ One participant had one event at day 2 and day 7, and two participants had two events each at day 7.

Table 2 summarizes participant satisfaction with the circumcision procedure and follow-up care received as well as resumption of work or normal activities at two and seven days post-circumcision. More than 95% of participants reported being very satisfied or somewhat satisfied with the results of the procedure and the follow-up care they received. One participant reported being very dissatisfied with the procedure and two were very dissatisfied with the quality of care they received during follow-up. Of note, the individual who was very dissatisfied with his procedure was found to have insufficient skin removed of moderate severity. After two days, slightly less than one-half (45%; Table 2) of participants had resumed normal activities and work whereas 74% reported returning to work after seven days.

**Table 2:** Satisfaction with circumcision procedure and follow-up at 2 and 7 days post-circumcision among HIV-uninfected, sexually-active adult men circumcised within the National Safe Male Circumcision program in Gaborone, Botswana.

		Days post-circumcision			
		Day 2 (n = 400)^a^		Day 7 (n = 395)	
Outcome		N	(%)	N	(%)
Satisfaction with circumcision procedure					
	Very satisfied	378	(94.5)	362	(95.3)
	Somewhat satisfied	16	(4.0)	21	(5.3)
	Somewhat dissatisfied	6	(1.5)	11	(2.8)
	Very dissatisfied	0	(0.0)	1	(0.3)
Satisfaction with follow-up care					
	Very satisfied	381	(95.3)	376	(95.2)
	Somewhat satisfied	11	(2.8)	13	(3.3)
	Somewhat dissatisfied	6	(1.5)	6	(1.5)
	Very dissatisfied	2	(0.5)	0	(0.0)
Resumed normal activities and/or work		181	(45.3)	291	(73.7)

^a^ One participant completed the clinical examination at the follow-up visit at day 2, but did not complete the satisfaction questionnaire.

## Discussion

In this prospective, clinic-based cohort study of adult men undergoing VMMC in southeastern Botswana, we observed an overall AE rate (excluding all pain-related complications) of 6.7%. The most common AE was hematoma, followed by infection and bleeding. The overwhelming majority of participants were very or somewhat satisfied with the circumcision procedure and the follow-up care they received. More than three-quarters of participants resumed normal activities seven days post-operative.

Although we found an overall AE rate that was approximately twice that observed in the trials, the rate was consistent with previously published reports with similarly high retention. A 2015 evaluation by Reed and colleagues of 1,699 surgically circumcised men in the Nyanza Province, Kenya within the public sector (of whom 94.2% were assessed for post-operative complications) reported an overall AE rate of 5.1% [10]. Studies conducted in other countries have reported lower AE rates (i.e. <2%) but suffered from poor retention [6, 9,14,15,19,20]. For example, an evaluation of a school-based VMMC campaign in South Africa reported an overall AE rate of 1.2%. Yet, only 64% and 50% of those circumcised were examined for complications at two and seven days post-circumcision, respectively [9]. VMMC programs with sub-optimal post-procedure follow-up may be missing complications that warrant medical attention [10].

A strength of this study was the high retention rate (97%), achieved by a combination of activities including providing participants with wallet-sized appointment reminder cards, telephone calls prior to each appointment, a financial incentive for each completed follow-up visit and in the event of a missed visit, up to three telephone calls and one home visit. Another strength was the data from a real-world setting, rather than a clinical trial setting, which likely closely represent the experience of mature, government-run urban VMMC clinic. While study staff worked closely with site clinicians to share all patient safety data in real time, they did not perform any circumcision procedures or directly provide post-procedure clinical care.

Our study is also subject to limitations. We restricted enrollment to men who resided within 25 km of our study sites (both of which were located in Gaborone, the capital city of Botswana and one of two major urban areas in the country). This inclusion criterion likely contributed to our very high retention rate (97%) but results may not generalize to men undergoing VMMC in rural or remote areas. We also provided reimbursement to participants, which may not be feasible in resource-constrained settings. Additionally, our findings do not apply to non-surgical circumcision with devices, such as the PrePex device. Unlike surgical techniques, devices do not require injectable anesthesia and can be applied by non-physician personnel in non-sterile settings. The Botswana Ministry of Health began offering the PrePex procedure to adult men in August 2014. However, VMMC programs must maintain a surgical option given that 5–7% of adult men and up to 53% of adolescent boys are ineligible for the PrePex procedure due to anatomical reasons [21–26]. Additionally, most complications associated with the PrePex procedure must be resolved surgically [23, 27]. Finally, this study was not powered to detect a statistically significant difference in the AE rate as compared to either the rate reported in trial settings or a pre-specified target. Instead, we sought to provide descriptive data on the safety of VMMC in a ‘real world’ setting using active surveillance methods aimed at assessing *all* persons undergoing VMMC for a pre-specified list of complications potentially associated with VMMC.

In summary, while patient satisfaction was overwhelmingly positive, we observed a higher overall AE rate compared to that reported in trial settings. VMMC programs must improve post-procedure retention to ensure accurate monitoring and evaluation of patient safety. Wallet-sized appointment reminder cards and active tracing of men who do not return for follow-up care used in the current study may be effective strategies. As national VMMC programs grow, safety of services must be prioritized to ensure the success of this research-tested, evidence-based HIV prevention intervention.

## Supporting information

S1 Appendix Patient questionnaire administered at day 2 follow-up visit. (PDF)

S2 Appendix Adverse event data collection form used at day 2 follow-up visit. (PDF)

S3 Appendix Patient questionnaire administered at day 7 follow-up visit. (PDF)

S4 Appendix Adverse event data collection form used at day 7 follow-up visit. (PDF)
