# Supplementary material for: A prospective cohort study of safety and patient satisfaction of voluntary medical male circumcision in Botswana
Source: PLoS One. 2017 Nov 7;12(11):e0185904. doi: 10.1371/journal.pone.0185904 (PMC5675416; doi:10.1371/journal.pone.0185904)
Supplement: S1 Appendix — (PDF) [file pone.0185904.s001.pdf]

National Safe Male Circumcision Program in Botswana:
Assessing Changes in Sexual Behavior, Adverse Events, and Uptake of Services

Study ID

FOLLOW-UP QUESTIONNAIRE: DAY 2

Data Collector (initials)

Today's Date

D	D	M	M	Y	Y	Y	Y
				2	0		

Instructions:

"I am going to ask you some questions about your satisfaction with your recent circumcision procedure. Your answers to these questions will be used to help educate people about the recommended healing processes after circumcision.

Please answer the questions as honestly as you can and remember that we will follow strict procedures to keep the information you provide private.

Please remember that you do not have to answer any questions that you do not want to answer and you may discontinue the interview at any time. This form will not have your name anywhere on it. You will be identified by a number only. Please remember that there are no right or wrong answers. If you have any questions or do not understand what I am asking you at any time throughout this interview, please ask for clarification.

Do you have any questions before we begin?"

ANSWER ANY QUESTIONS AND ADDRESS RESPONDENT'S CONCERNS.

1	**How do you clean your wound?**<br>*How do you clean your wound?*	☐ SOAP AND WATER<br>☐ SALT WATER<br>☐ TRADITIONAL MEDICINE<br>☐ SPIRITS<br>☐ OTHER (specify) ____________________<br>____________________<br>☐ PREFER NOT TO ANSWER	
2	**How would you report your satisfaction with the circumcision procedure?**<br>*How would you report your satisfaction with the circumcision procedure?*	☐ VERY SATISFIED<br>☐ SOMEWHAT SATISFIED<br>☐ SOMEWHAT DISSATISFIED<br>☐ VERY DISSATISFIED<br>☐ PREFER NOT TO ANSWER	**SKIP TO 4** (VERY SATISFIED, SOMEWHAT SATISFIED)
3	**Why are you dissatisfied with the circumcision procedure? *(Select all that apply)***<br>*Why are you dissatisfied with the circumcision procedure?*	☐ OVERALL APPEARANCE<br>☐ WOUND CARE REQUIREMENTS<br>☐ COMPLICATION RELATED TO THE PROCEDURE<br>☐ OTHER (specify) ____________________<br>____________________<br>☐ PREFER NOT TO ANSWER	

Study ID

National Safe Male Circumcision Program in Botswana:
Assessing Changes in Sexual Behavior, Adverse Events, and Uptake of Services

4	**How would you report your satisfaction with circumcision follow-up care?**<br>*How would you report your satisfaction with circumcision follow-up care?*	☐ VERY SATISFIED<br>☐ SOMEWHAT SATISFIED<br>☐ SOMEWHAT DISSATISFIED<br>☐ VERY DISSATISFIED<br>☐ PREFER NOT TO ANSWER	**SKIP TO 6** (VERY SATISFIED, SOMEWHAT SATISFIED)
5	**Why are you dissatisfied with follow-up care? *(Select all that apply)***<br>*Why are you dissatisfied with follow-up care?*	☐ WAIT TIME AT CLINIC<br>☐ PROVIDER SKILL LEVEL<br>☐ TRANSPORT DIFFICULTIES<br>☐ OTHER (specify)<br>____________________<br>____________________<br>____________________<br>☐ PREFER NOT TO ANSWER	
6	**Have you resumed normal activities or work since the circumcision procedure?**<br>*Have you performed normal activities or work since the circumcision procedure?*	☐ YES<br>☐ NO<br>☐ PREFER NOT TO ANSWER	**SKIP TO 8** (NO, PREFER NOT TO ANSWER)
7	**How many days after the circumcision procedure did you resume normal activities or work?**<br>*How many days after the circumcision procedure did you resume normal activities or work?*	______ DAYS<br>☐ PREFER NOT TO ANSWER	
8	**Have you had an erection since the circumcision procedure?**<br>*Have you had an erection since the circumcision procedure?*	☐ YES<br>☐ NO<br>☐ PREFER NOT TO ANSWER	**SKIP TO 10** (NO, PREFER NOT TO ANSWER)
9	**Did the erection cause any of the following?**<br>*Did the erection cause any of the following?*<br>*(check all that apply)*	☐ PAIN<br>☐ BLEEDING<br>☐ OPENING UP OF THE WOUND<br>☐ WEAK ERECTION<br>☐ OTHER (specify)<br>____________________<br>____________________<br>☐ PREFER NOT TO ANSWER	
10	**Have you had sexual intercourse since the circumcision?**<br>*Have you had sexual intercourse since the circumcision?*	☐ YES<br>☐ NO<br>☐ PREFER NOT TO ANSWER	**FINISHED** (NO, PREFER NOT TO ANSWER)
11	**How often did you use a condom during sexual intercourse since the circumcision procedure?**<br>*How often did you use a condom during sexual intercourse since the circumcision procedure?*	☐ NEVER<br>☐ SOMETIMES<br>☐ OFTEN<br>☐ ALWAYS<br>☐ DON'T KNOW<br>☐ PREFER NOT TO ANSWER	

Study ID

