# Supplementary material for: A prospective cohort study of safety and patient satisfaction of voluntary medical male circumcision in Botswana
Source: PLoS One. 2017 Nov 7;12(11):e0185904. doi: 10.1371/journal.pone.0185904 (PMC5675416; doi:10.1371/journal.pone.0185904)
Supplement: S2 Appendix — (PDF) [file pone.0185904.s002.pdf]

National Safe Male Circumcision Program in Botswana:
Assessing Changes in Sexual Behavior, Adverse Events, and Uptake of Services

Study ID

CLINICAL EXAM TOOL: DAY 2

Clinician (initials)

Today's Date

D	D	M	M	Y	Y	Y	Y
				2	0		

ADVERSE EVENTS

Instructions: *The following table lists adverse events that may occur at this time, and provides definitions of severity. Inspect the client's penis and determine the severity of present signs and symptoms based upon the definitions provided. Relationship of the adverse event to the circumcision procedure should also be recorded. The following definitions should be used:*

Not likely related: A complication has occurred that is clearly explained by a cause other than the circumcision procedure

Possibly related: A complication has occurred during or after surgery that could be explained by the circumcision procedure, but can equally or more likely be explained by other causes

Probably related: A complication has occurred during or after surgery that is most likely due to the circumcision procedure, but could be explained by other causes

Definitely related: A clear complication of the procedure has occurred either during or after the procedure

	DESCRIPTION OF ADVERSE EVENT	SEVERITY	RELATED TO CIRCUMCISION
**HEMATOMA**		☐ NOT PRESENT	☐ NOT LIKELY<br>☐ POSSIBLE<br>☐ PROBABLE<br>☐ DEFINITELY
	More swelling than usual, but no significant discomfort	☐ MILD	
	Significant tenderness & discomfort, but surgery not required	☐ MODERATE	
	Surgical re-exploration required to correct	☐ SEVERE	
**BLEEDING**		☐ NOT PRESENT	☐ NOT LIKELY<br>☐ POSSIBLE<br>☐ PROBABLE<br>☐ DEFINITELY
	Dressing soaked through with blood at a routine follow-up visit	☐ MILD	
	Bleeding that requires a special return to the clinic for medical attention	☐ MODERATE	
	Bleeding that requires surgical re-exploration	☐ SEVERE	
**INFECTION**		☐ NOT PRESENT	☐ NOT LIKELY<br>☐ POSSIBLE<br>☐ PROBABLE<br>☐ DEFINITELY
	Erythema more than 1cm beyond incision line	☐ MILD	
	Purulent discharge from wound	☐ MODERATE	
	Cellulitis or wound necrosis	☐ SEVERE	
**PAIN**		☐ NOT PRESENT	☐ NOT LIKELY<br>☐ POSSIBLE<br>☐ PROBABLE<br>☐ DEFINITELY
	3-4 on pain scale	☐ MILD	
	5-6 on pain scale	☐ MODERATE	
	7 and greater on pain scale	☐ SEVERE	
**DAMAGE TO PENIS**		☐ NOT PRESENT	☐ NOT LIKELY<br>☐ POSSIBLE<br>☐ PROBABLE<br>☐ DEFINITELY
	Mild bruising or abrasion, no further treatment necessary	☐ MILD	
	Bruise or abrasion to the glans or shaft requiring pressure dressing or additional surgery to control	☐ MODERATE	
	Portion or all of the glans or shaft of penis severed	☐ SEVERE	

National Safe Male Circumcision Program in Botswana:
Assessing Changes in Sexual Behavior, Adverse Events, and Uptake of Services

Study ID

	DESCRIPTION OF ADVERSE EVENT	SEVERITY	RELATED TO CIRCUMCISION
**EXCESSIVE SKIN REMOVED**		☐ NOT PRESENT	☐ NOT LIKELY<br>☐ POSSIBLE<br>☐ PROBABLE<br>☐ DEFINITELY
	No discernible abnormality, but client concerned	☐ MILD	
	Skin is tight, but no further surgery necessary	☐ MODERATE	
	Requires operation or transfer to another facility to correct problem	☐ SEVERE	
	Participant circumcised with PrePex device that is still intact	☐ NOT APPLICABLE	
**INSUFFICIENT SKIN REMOVED**		☐ NOT PRESENT	☐ NOT LIKELY<br>☐ POSSIBLE<br>☐ PROBABLE<br>☐ DEFINITELY
	Prepuce partially covers the glans only when extended	☐ MILD	
	Prepuce still partially covers glans and operation is required to correct	☐ MODERATE	
	Not applicable	☐ SEVERE	
	Participant circumcised with PrePex device that is still intact	☐ NOT APPLICABLE	
**DELAYED WOUND HEALING**		☐ NOT PRESENT	☐ NOT LIKELY<br>☐ POSSIBLE<br>☐ PROBABLE<br>☐ DEFINITELY
	Healing taking longer than usual, but no extra treatment necessary	☐ MILD	
	Additional non-surgical treatment required	☐ MODERATE	
	Requires surgery to correct	☐ SEVERE	
**ANESTHETIC-RELATED EVENT**		☐ NOT PRESENT	☐ NOT LIKELY<br>☐ POSSIBLE<br>☐ PROBABLE<br>☐ DEFINITELY
	Palpitations, vaso-vagal reaction or emesis	☐ MILD	
	Reaction to anesthetic requiring medical treatment in study clinic but not transfer to another facility	☐ MODERATE	
	Anaphylaxis or any reaction requiring transfer to another facility	☐ SEVERE	
**VOIDING PROBLEMS**		☐ NOT PRESENT	☐ NOT LIKELY<br>☐ POSSIBLE<br>☐ PROBABLE<br>☐ DEFINITELY
	Transient complaint that that resolves without treatment	☐ MILD	
	Requires a special return to the clinic, but no additional treatment	☐ MODERATE	
	Requires referral to other facility or specialist for management	☐ SEVERE	
**EARLY REMOVAL**	Did the participant return with the device components intact and request early removal of the device before the scheduled removal day?	☐ YES<br>☐ NO<br>☐ NOT APPLICABLE	☐ NOT LIKELY<br>☐ POSSIBLE<br>☐ PROBABLE<br>☐ DEFINITELY
**DEVICE DISPLACEMENT**	Is the device displaced or partially detached?	☐ YES<br>☐ NO<br>☐ NOT APPLICABLE	☐ NOT LIKELY<br>☐ POSSIBLE<br>☐ PROBABLE<br>☐ DEFINITELY
**SELF REMOVAL OF DEVICE**	Was the device removed by the participant?	☐ YES<br>☐ NO<br>☐ NOT APPLICABLE	☐ NOT LIKELY<br>☐ POSSIBLE<br>☐ PROBABLE<br>☐ DEFINITELY
**DEVICE MALFUNCTION**	Did any of the components of the device malfunction?	☐ YES<br>☐ NO<br>☐ NOT APPLICABLE	☐ NOT LIKELY<br>☐ POSSIBLE<br>☐ PROBABLE<br>☐ DEFINITELY
**DIFFICULTY IN REMOVING DEVICE**	Was there difficulty with removal of the device?	☐ YES<br>☐ NO<br>☐ NOT APPLICABLE	☐ NOT LIKELY<br>☐ POSSIBLE<br>☐ PROBABLE<br>☐ DEFINITELY

**Is the client currently experiencing an <u>adverse event</u>?**<br>*YES=at least one moderate or severe adverse event is present*<br>*NO=the healing and symptoms are within normal range*	☐ YES →<br>☐ NO	**NOTIFY STUDY COORDINATOR IMMEDIATELY**

National Safe Male Circumcision Program in Botswana:
Assessing Changes in Sexual Behavior, Adverse Events, and Uptake of Services

Study ID

STI ASSESSMENT		
**Instructions:** *Examine the penis and document in the table below whether the client exhibits any of the following conditions.*		
• Temp > 38°C	☐ YES	☐ NO
• Urethral discharge	☐ YES	☐ NO
• Genital sore or ulcer	☐ YES	☐ NO
• Vesicles	☐ YES	☐ NO
• Abdominal mass	☐ YES	☐ NO
• Rebound tenderness, guarding	☐ YES	☐ NO
• Scrotal swelling, pain	☐ YES	☐ NO
• Elevated/rotated testes	☐ YES	☐ NO
